# The Impact of a Non-profit Market on Food Store Choice and Shopping Experience: A Community Case Study

**DOI:** 10.3389/fpubh.2019.00078

**Published:** 2019-04-08

**Authors:** Mengni Yao, Amy Hillier, Elizabeth Wall, Katherine I. DiSantis

**Affiliations:** ^1^School of Social Work, Boston University, Boston, MA, United States; ^2^School of Social Policy & Practice, University of Pennsylvania, Philadelphia, PA, United States; ^3^Perelman School of Medicine, University of Pennsylvania, Philadelphia, PA, United States; ^4^College of Health Sciences, Arcadia University, Glenside, PA, United States

**Keywords:** food desert, supermarket, food shopping behaviors, food access, non-profit supermarket

## Abstract

Research evaluating the impact of new food stores in “food deserts” have reported limited impact on eating and health outcomes of residents who live nearby. Few studies have reported on shoppers' food store choices and experiences in these new stores. This study focused on residents' experience with a new non-profit food market in Chester, PA and analyzes spatial patterns regarding who did and did not choose to shop at the new store. Phone surveys (*n* = 135) and in-person interviews (*n* = 13) were conducted with the primary food shopper for households living in Chester 1–2 years, respectively, after the opening of a store. Participants who shopped at the new market reported positive experiences in regard to convenience, customer service, food quality, and prices and believed that the new market had a positive impact on the community. But most participants had not shopped at the new market, citing many of the same factors in their decision to shop at supermarkets outside the city. Our findings underscore the need to combine new food retail strategies with community engagement and other interventions, such as in-store promotions and health education programs, to maximize the number of people who shop at new food outlets designed to improve access to healthful foods.

## Introduction

A wide range of public health studies have emphasized the importance of local food environments on the prevalence of chronic, diet related illness. Low-income and minority communities often lack access to healthy, affordable foods (1, 2), and living in these “food deserts” has been shown to be associated with health disparities (3, 4) Some recent research has challenged this connection between food environment and health outcomes (5–8), but the evidence has been sufficient to motivate a range of interventions aimed at increasing physical access to healthful foods (9–11). Most prominent among these interventions have been new supermarkets in former “food deserts” (12, 13). Most of these new stores follow the format of conventional for-profit full-service supermarkets. In this study, we examine the experience of residents within a food desert with a novel non-profit food market.

## Background and Rationale

Only a limited number of studies have evaluated the impact of new supermarkets on eating and health outcomes for residents who live nearby, and they show modest results. While one published study found significant positive impact on perception of the food environment (14), the majority of studies found that new markets in food deserts are not associated with significant changes in dietary intake or body mass index (BMI) (15, 16). Two other studies that found an association between the opening of a new store and changes in diet were not able to link those changes directly to use of the new food store (17, 18). Evaluations of new supermarkets have also reported varying levels of adoption of the market. One found that only 26.7% of residents chose the new store as their primary food shopping destination (17) whereas, another identified 68.3% residents as regular users of their new market (18). In order to better understand the role of new stores in former food deserts, it is crucial to understand food store choice as well as residents' experiences in the food retail environment (19).

Research has shown that food shopping behaviors relate to social dynamics, including socioeconomic status, family responsibilities, and practical needs, and the social environment, including cues about personal safety and belonging, not just distance and the physical environment (20). Low-income minority women in one study identified material, economic and social-interactional barriers to accessing healthy food (21), while Black and Latino participants in another study noted that price, transportation and Supplemental Nutrition Assistance Program (SNAP) benefits were key factors for their shopping choices (22). Another study found that participants appreciated the new market for helping them cope with difficult shopping routines and for providing a reliable high-quality option with a healthy physical and social environment (23). Together these studies show that, in addition to food price, selection, and quality, social factors matter and different people experience store choices differently. Qualitative or mixed-methods research is particularly useful for understanding these issues.

Through this community case study, we focus on the initial step in the causal process linking food shopping and health—the decision about where to shop for food. By considering the experience of residents who shopped at a new non-profit market in a former food desert in Chester, Pennsylvania as well the reasons cited by other residents about why they chose not to shop there, we seek to contribute to understanding about the relationship between physical proximity to food stores and food store choice.

## Setting

Chester, PA is a city of approximately 34,000 residents, 70.4% of whom are Black/African American and 34.6% of whom are living below the federal poverty line (24, 25). Health disparities are pronounced in Chester where the rates of obesity and diabetes are substantially higher than those in Delaware County (26). Following significant job loss and population decline related to deindustrialization in the second half of the twentieth century, Chester lost all of the supermarkets within its 6 square miles. Despite having no supermarkets, the Chester waterfront became home to a large casino in 2006 and state-of-the-art professional soccer stadium in 2010.

In September 2013, Philabundance, the region's largest food bank, opened a new non-profit market called Fare & Square in Chester. Philabundance had experience with a client choice food pantry in nearby Philadelphia but no prior retail experience. With support from federal, state, local, and foundation grants, Philabundance was able to purchase the site of a former supermarket at the west end of Chester, half of which housed a large dollar store that has remained open since the sale. Fare & Square moved in to the remaining 13,000 square feet. The new store featured a higher proportion of fresh foods than conventional for-profit supermarkets and the “Carrot Club” which allows low-income households to receive 3% benefits on food purchases as credit toward future store purchases. In most other ways, Fare & Square resembled a conventional large grocery store or small supermarket.

## Methods

In 2013, 715 adults who were the primary food shopper for their household and lived in Chester or one of the surrounding towns were recruited to complete surveys about their food shopping, eating, and health. Analyses for this study utilized only the follow-up data of the 135 participants who completed that assessment again in 2015; we report results of behavior change over time among study participants elsewhere (18) A small number of those who reported shopping at the new store during the first year (*n* = 13) also participated in semi-structured interviews about their food shopping experiences in December 2014. Participants received gift cards for participating in each phase of the study: $10 for the initial survey, $10 for the follow-up survey, and $50 for an interview.

Close-ended survey questions were analyzed using descriptive statistics in SPSS version 22. ArcGIS 10.2.2 was used to map and calculate the straight-line distance between participants' home and their primary supermarket choice. Interviews were audio recorded and transcribed by members of the research team. Open-ended survey responses and semi-structured interviews were coded based on emergent themes. A codebook was created after two members of the research team independently read and manually coded a subset of the transcripts. Using the codebook, the same two researchers coded all the transcripts using NVivo. These codes were then used to develop themes to organize the responses.

The study was approved by the University of Pennsylvania Institutional Review Board.

## Results

The majority of participants were women (*n* = 90) and Black/ African American (*n* = 114) with an average age of 52. Three quarters of participants were SNAP recipients (*n* = 102) while only a small portion of the individuals (10.4%) reported receiving WIC benefits. Less than half (37%) reported having shopped at Fare & Square in the previous year. See Table 1.

**Table 1:** Descriptive statistics from survey.

	**Total (*****N*** **=** **135) n%**		**Shopped at F&S (*****N*** **=** **50) n%**		**Did not shop at F&S (*****N*** **=** **85) n%**	
**SEX**						
Male	45	33.3	14	28.0	31	36.5
Female	90	66.7	36	72.0	54	63.5
**RACE**						
Black/ African American, not Hispanic	114	84.4	46	92.0	68	80
White/ Caucasian not Hispanic	12	8.9	3	6.0	9	10.6
Multi-racial	2	1.5			2	2.4
Other	7	5.2	1	2.0	6	7.1
**AGE**						
Mean	50.4		51.2		50	
Standard deviation	11.0		11.9		10.4	
WIC	14	10.4	6	12.0	8	9.4
SNAP	102	75.6	37	74.0	65	76.5
EDUCATION LEVEL^*^						
Some high school or less	24	17.8	8	16.0	16	18.8
High school graduate or GED certificate	69	51.1	27	54.0	42	49.4
Some college or technical school	20	14.8	7	14.0	13	15.3
College graduate or more	20	14.8	7	14.0	13	15.3
N/A	2	1.5	1	2.0	1	1.2
Children under 18	49	36.3	23	46.0	26	30.6
**VEHICLE**						
0	82	60.7	25	50.0	57	67.1
1	46	34.1	22	44.0	24	28.2
2	7	5.2	3	9.0	4	4.7
**INCOME LEVEL**						
Don't know	22	16.3	8	16.0	14	16.5
<$15,000	71	52.6	30	60.0	41	48.2
$15,000-$24,999	24	17.8	5	10.0	19	22.4
$25,000-$49,999	14	10.4	6	12.0	8	9.4
$50,000 or more	4	3.0	1	2.0	3	3.5
**EMPLOYMENT**						
None	81	60.0	32	64.0	49	57.6
Up to 20 h	15	11.1	8	5.9	7	8.2
More than 20 but <35 h	17	12.6	3	6.0	14	16.5
More than 35 h	22	16.3	6	12.0	16	18.8

* *Two participants failed to answer this question*.

Interview participants had similar characteristics to survey participants. Nearly all (92%) identified as female and their ages ranged from 39 to 64. They were predominantly African American (92%) with 8% identified as Hispanic. Most participants (77%) reported receiving SNAP benefits.

To better understand participants' shopping experiences, we combined responses from the close-ended and open-ended survey questions with the in-depth interviews.

### The Experience of Shopping at Fare and Square

The reasons participants had for choosing Fare & Square varied as did their experiences of shopping there. Convenience emerged as a primary consideration and characterized the experience of many survey and interview participants. The other main factors were price, selection and quality of food, quality of food environment, social dynamics, and community impact.

### Convenience

Participants consistently referenced greater convenience for themselves and other Chester residents as a positive aspect of Fare & Square opening. The majority of participants (76%) who had shopped at Fare & Square the previous year indicated that it was more convenient for them to shop there than the other supermarkets in the area. For some people, this was about physical proximity. Participants who lived at the west end of Chester, near Fare & Square, often found it easy to drive or even walk to the store.

**Figure 1:**
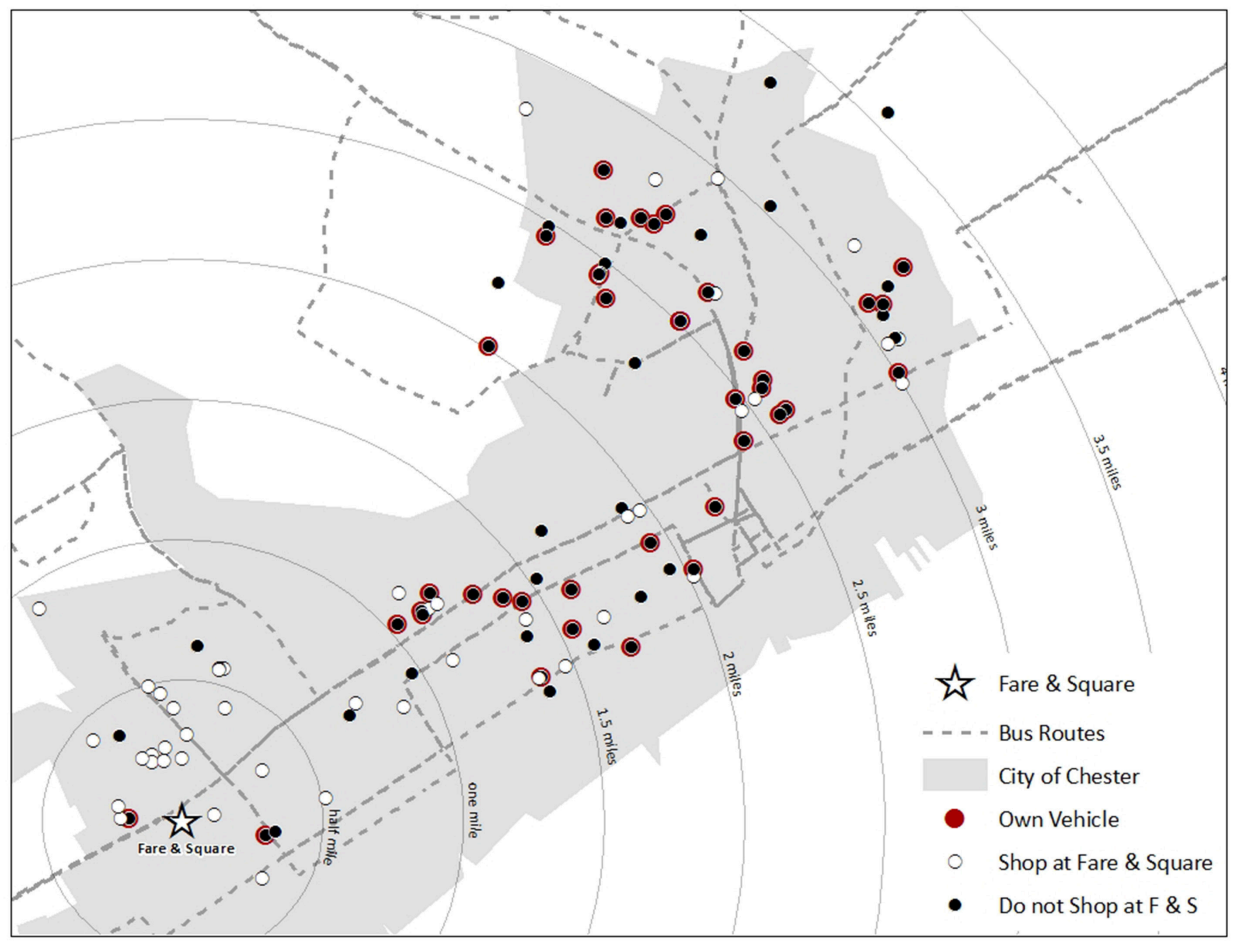
Map of study participants and car ownership.

Figure 1 shows that most participants who lived within a half mile of Fare & Square had shopped there in the previous year. “A lot of people… don't have the transportation to get out of Chester. [Fare & Square] is more convenient if you don't have your own car,” explained a survey participant. And other participants who didn't live close, themselves, said that Fare & Square was more convenient for others, particularly seniors and people without cars. “The people, especially senior citizens that live down on that end of the city… benefit because it's easy to get there… They can walk.”

For others, Fare & Square was convenient because it was easily accessible by bus. Figure 1 shows that a number of participants who did not own vehicles and live along the bus line connecting the east and west ends of Chester shopped at Fare & Square. “The bus drops you right off at the door,” noted one survey participant. Convenience also meant being close to work or other places where participants spent time. “I usually stay down this end… cause of my work schedule…Cause if I'm pressing for time, if I have an appointment, I usually stick right there, because Fare & Square is my best bet,” said one interview participant. “I benefit because I go to church close to Fare & Square so I go grocery shopping after church before I go home,” explained a survey respondent.

Convenience meant more than just physical proximity; it also meant having more control over where and when someone did their food shopping. By having a market within walking distance or easily accessible by bus, residents felt a greater sense of independence, particularly for those who were dependent upon friends and family for a ride before Fare & Square opened. “I had to pay people to take me and I had to go on their time so I could only go to certain stores and that most of time it was a convenience store or corner store or something. So I didn't… have money to spend like that so it was very, very difficult,” said one interview participant. Explained another interview participant, “It definitely has less stress wondering how I'm going to get to a market, or depending on someone else for a ride, so it's definitely made a change in my life.” Bus fares and paying others with cars to drive them added to the financial burden of food shopping before the opening of Fare & Square.

### Price

For those who shopped at Fare & Square, especially participants who were low-income and received SNAP or WIC benefits, shopping at Fare & Square made budgeting for the month easier. “I benefit because I can get more groceries because of the reasonable prices,” said one survey participant. “Everybody that goes there and shops, they benefit because the prices are good, and the store is laid out easy to find everything you need.” Participants also benefitted from Fare & Square's Carrot Cash program. Nearly all (92%) of the participants who had shopped at Fare & Square were Carrot Cash members. “I benefit because of the carrot cash helps me when I run out of food.”

### Food Quality and Selection

Fare & Square increased access to fresh food, a welcome change from the packaged and processed foods sold at smaller corner stores. “It's bringing fresh fruits and vegetables which we may not have, into the city,” explained one interview participant. “You'd might be surprised how many kids think vegetables get out of a can.” This same participant also mentioned that before the store opened, many children in the community had never seen fresh fish before, thinking that fish comes from a plastic bag.

Participants commented that the food sold at Fare & Square is “up to date,” not expired or “all beat up and damaged.” Said one interview participant, “They have like a good quality of food, it's not just something that was thrown here; they actually give out good products.” Explained a survey participant, “My friends and neighbors that live near Fare & Square benefit by having better access to fresh fruits and produce.” Fare & Square has helped to raise the expectation and sense of deserving among Chester residents by providing quality food.

According to the survey results, 72% of the 50 participants who frequented Fare & Square buy fresh vegetables there. Five participants were able to buy different kinds of foods that they had not bought before the store opened, including new types of vegetables and tropical fruits, and Caribbean syrup. Access to fresh and high-quality foods was particularly important to people managing chronic health conditions and who had special dietary needs. The majority of those who had shopped at Fare & Square (62%) expressed that shopping there made it easier for them to buy food they were supposed to eat to manage their health. Others cited the availability of culturally appropriate foods as a benefit. “They have the products especially the Spanish foods,” explained one survey participant.

### Physical Environment

Participants also spoke appreciatively about how clean and organized Fare & Square is. “It's nice and clean in here… you can see what you want, you don't have to really fumble around for nothing,” said one interview participant. “When I go to the other markets… they're kinda congested at times and stuff is everywhere sometimes,” explained another interview participant. Most participants felt safe shopping at Fare & Square and appreciated the safety measures that the store has taken. “I definitely feel safe, they have security… and even if they don't have security… if I feel uncomfortable I could ask one of the staff members, ‘Could you walk me outside?’”

### Community Impact

Many participants emphasized that in addition to increased convenience and higher quality foods and physical environment, Fare & Square has had a positive community impact on Chester. Living in the city with high poverty, residents appreciate that Fare & Square provides great customer service, hires local residents, and hosts health promotion programs.

Among those who had shopped at Fare & Square, 56% agreed that the customer service at the store was better than that at other supermarkets in the area. “The security people, they greet you when you come in. There's always people out to help you on the floor and things. It's very, it's a very friendly atmosphere,” said one interview participant. “People are friendly here. When you go in the bigger markets, they don't have patience.” Another interview participant said that “it feels good” to be recognized by store staff. “They know me by name, they're awesome.”

The majority (80%) of those who had shopped at Fare & Square said that they frequently run into people they know while they were in the store, including employees. “I'm glad to see that they have … employed people from the neighborhood, and they were keeping their jobs… they normally hire people from out of the town and I don't think it's fair.” Another interview participant described it as “a reunion store, like everybody would meet up here. Like I said, people you haven't seen for years that you went the school with.” This contributed to the sense of belonging and ownership. “There is just a sense of pride cause this is our market,” explained an interview participant. Sponsoring health promotion programs, such as free mammograms, nutrition education and cooking classes, is another way Fare & Square serves the community.

### The Reasons for Not Shopping at Fare and Square

The majority of survey participants (63.0%) had not shopped at Fare & Square in the previous year, and even among those who had shopped at Fare & Square, most also shopped at other food stores. Many of the same themes as those cited by participants who did choose to shop at Fare & Square—convenience, price, food selection—emerged as explanations of their store choice. Participants experienced and perceived these decision factors differently based on personal and family preferences and their own expectations and previous shopping patterns. Store size and perception of safety were also cited as factors.

### Convenience and Transportation

As the largest food market in Chester, Fare and Square is convenient for those who live close by. However, Fare & Square is not necessarily the go-to market for residents who live on the opposite side of the city and may be physically closer to suburban supermarkets. One survey participant explained, “I've never been there. I forget it's over on the other side of town. We just go to stores closer to our side of town.” Another survey participant explained that the store is “so far down at the other end of town, it's too far for me, I don't have a car.” Some perceived public transportation to be challenging and time-consuming. “I have to catch two buses to get there… it's not convenient.” Geographical distance and car ownership influenced decision-making for grocery shopping. Many live closer to food stores other than Fare & Square even though those stores are located outside Chester. Participants who live near Fare & Square and own a car often chose to travel to supermarkets outside of Chester rather than relying on Fare & Square.

### Perception of Safety

Participants had mixed reviews regarding the safety of the area around Fare & Square. The store was located on the west side of the city, and those who shopped at the store had familiarity with this part of the city. Those shoppers who were not from the west side tended to raise concerns about the location included saying it was location in a “rough neighborhood” and that it was “too far and scary.” Even some who shopped at Fare & Square expressed concern about shopping there at night. “I try to get out in the morning or in the afternoon so that I can get back in light hours,” she explained. “This is…really getting bad now.”

Another interview participant who shopped at Fare & Square worried about the lighting around the side and back of the store. “See, you got this big parking lot in the back, and then y'all ain't got lights, like the street lights… and if you come down here in the evening when they get dark, a lot of times you'll be skeptical about turning in here because you don't have enough lighting.”

### Price and Promotion of Sales

There were also mixed reviews about the prices at Fare & Squares. Participants compared prices among stores, often shopping outside Chester or even outside Pennsylvania, in nearby Delaware, to save money. Fare & Square lowered its prices shortly after opening, but participants who shopped right after it opened made reference to the initial prices that they perceived to be high relative to other stores. One survey participant described the prices as “outrageous,” commenting, “Stuff on sale is good but everything else is too much.” Others cited the cost of specific foods, including meat and fresh fruit. One survey participant described being unclear of the price promotion patterns at the new market and that this confusion led to her not shopping there at times and said that “I haven't had the money to go, because I go to other stores when I see they have sales. I don't know when Fare & square has sales.”

### Store Size and Food Selection

Participants also made reference to the relatively small size of Fare & Square, which was often discussed with a negative connotation. “When I come here, just when I look around, it…feels like I'm going to a regular, round the neighborhood store, it's like small… Put some more stuff that make it look like it's a market instead of a corner store,” explained one interview participant. “I don't feel like it's a real market. It's more like a large convenience store,” said one survey participant. The limited food selection, a byproduct of the store size, was another concern shared by many participants and they had to make additional trips to other stores. One survey participant complained about the “small, bad selection… When I went there, I didn't see what I was looking for.” Others suggested “more name brand things” and “more variety and more meat products.” Another said that “it is kind of congested” and the store could use “more space and more lighting.”

## Discussion

This study illustrates that participants who had shopped at Fare & Square largely had positive experiences. They perceived that the new market benefitted themselves and other Chester residents by increasing convenience, providing high quality fresh produce, offering competitive prices and excellent customer service, and creating positive impact within community. Increased convenience was especially important for the elderly and families who lived in close proximity to the store who no longer had to rely on public transportation or others for rides to do their grocery shopping. The availability of high quality and fresh foods at a store located within the city served as a source of pride as well as health. Seeing friends, relatives and community leaders shopping and working at the store fostered a sense of community ownership.

But despite the positive experiences of those who shopped at Fare and Square, most participants had not shopped there in the previous year even though they lived in or near Chester. And the majority of participants—even those who did shop at Fare & Square—purchased food items from multiple stores. This was true before Fare & Square was built and remained true after it opened. These results confirm what other studies have found: distance matters, especially for those without a vehicle, but residents are often motivated to travel beyond the closest market and to utilize multiple markets to meet their families' needs. Participants who did shop at Fare & Square and those who did not cited the same factors in their decision-making; nevertheless, they experienced those differently. Overall, participants who lived on the east side of Chester, further from Fare & Square, preferred larger chain supermarkets outside the city.

Our findings highlight the fact that building a new food market—even one with high quality and reasonably-priced foods and offering good customer service—is no guarantee that residents will shop there. Additionally, this study reveals that new subsidized food markets in food deserts may not be able to compete with larger full-service chain supermarkets even when the new markets are closer to residents. Unless residents choose to shop at a new store and change the foods they purchase, it is not reasonable to expect new store interventions to improve health outcomes. The results from this study are consistent with findings of other studies from Philadelphia and Pittsburgh (17, 18). Large full-service supermarkets with good prices near where people live or spend time are most likely to attract the most customers, but by themselves, new supermarkets are likely not enough to change food shopping behaviors widely and improve health outcomes at a population level. If these new subsidized stores are going to positively impact the health of residents, it is critical for residents to shop at them regularly if not exclusively. In order to accomplish that, new stores need to attract customers by adding other health and social services, such as health screening, cooking classes, and nutrition education. Furthermore, these additional health promoting services could influence food item choice which in turn could positively impact health outcomes. Strong relationships between the store and surrounding community are also essential to long-term success.

This study evaluated the impact of a novel non-profit food market on food shopping in a former food desert. Using qualitative interviews and close-ended and open-ended survey questions, it offers insight into the experience of shopping at a new food store as well as reasons residents chose to shop elsewhere. This study is notable for following up with participants 2 years after the opening of the store while other studies have given supermarket interventions less than a year to demonstrate impact (17, 18). The maps and spatial analysis also emphasize the importance of spatial relationships for food store choice even though distance was not the primary or exclusive concern. This study is also notable in describing the experience of a large non-profit organization creating and sustaining a non-profit food market. We should note that in April 2018, Philabundance announced that they were turning Fare & Square over to a local for-profit supermarket chain. We recommend that any other non-profits considering opening a food store carefully study this case study before embarking on such an investment. They should consider the relationship of the non-profit to the surrounding community, the initial and long-term operating costs, and the organizations' retail experience.

These strengths of the study and the findings should be considered in light of the following limitations. The convenience nature of the sample limits the generalizability. The sample size decreased significantly from baseline to follow-up, reflecting limited funding available to track participants over the 2-year study time frame. The maps in this study only show stores where participants shopped for food most frequently even though most participants shopped at multiple stores. There was potential social response bias because of differences in the racial, age and economic status between researchers and most participants. While we did ask if participants had a vehicle, there were no survey questions about the transportation methods they actually used for food shopping.

## Conclusion

Future research should employ longitudinal analysis and mixed-methods to understand choices about food store shopping and food item choice. By looking over a longer period of time at what residents in former food deserts are buying, and how that changes with new supermarkets, researchers would be more likely to see measurable long-term health benefits. This requires multiple measures of health behaviors, including food store choice, food purchases, and food consumption, over multiple points in time. If new stores are incorporating creative, healthful in-store marketing and nutrition education programs, those should be evaluated, as well. Additionally, more research studies from different cities with larger sample size are encouraged since they can increase the generalizability of the study to achieve the goal of truly understanding health behavior change and addressing diet-related chronic disease. Finally, future research should consider the relationship between urban agriculture efforts and locally-owned and operated food retail outlets through the lens of food sovereignty, recognizing that food retail is just one part of urban food systems.

## Ethics Statement

This study was carried out in accordance with the recommendations of the University of Pennsylvania Institutional Review Board with written informed consent from all subjects. All subjects gave written informed consent in accordance with the Declaration of Helsinki. The protocol was approved by the University of Pennsylvania Institutional Review Board.

## Author Contributions

MY conducted the quantitative and spatial data analysis, and drafted the manuscript. AH provided assistance on data analysis and contributed to manuscript revisions. EW conducted the initial qualitative data analysis. KD contributed to manuscript revisions.

### Conflict of Interest Statement

The authors declare that the research was conducted in the absence of any commercial or financial relationships that could be construed as a potential conflict of interest.
